# Single-particle virology

**DOI:** 10.1007/s12551-020-00747-9

**Published:** 2020-09-03

**Authors:** Bálint Kiss, Dorottya Mudra, György Török, Zsolt Mártonfalvi, Gabriella Csík, Levente Herényi, Miklós Kellermayer

**Affiliations:** Department of Biophysics and Radiation Biology, Semmelweis University, Budapest, Hungary

**Keywords:** Single-molecule mechanics, Atomic force microscopy, Optical tweezers, Super-resolution microscopy, Viral genome packaging, Viral genome release

## Abstract

The development of advanced experimental methodologies, such as optical tweezers, scanning-probe and super-resolved optical microscopies, has led to the evolution of single-molecule biophysics, a field of science that allows direct access to the mechanistic detail of biomolecular structure and function. The extension of single-molecule methods to the investigation of particles such as viruses permits unprecedented insights into the behavior of supramolecular assemblies. Here we address the scope of viral exploration at the level of individual particles. In an era of increased awareness towards virology, single-particle approaches are expected to facilitate the in-depth understanding, and hence combating, of viral diseases.

## Single-molecule and single-particle science

Much of our knowledge in natural sciences is derived from ensembles of particles (atoms, molecules), the standard quantity of which is the mol (Van Holde et al. 1998). The properties and the behavior of the individual particles are thus extrapolations from ensemble average parameters. By contrast, single-molecule and single-particle science focuses on the individual (Bustamante et al. 2000; Kellermayer 2005). Hence, not only the average but also the distribution of the measured parameter can be obtained, which provides a direct insight into the structure, function, and dynamics of the investigated molecule or particle and into the mechanisms behind the processes the molecule or the particle is involved in. Investigation of molecules and particles one by one has particular significance in biological systems, considering that in a living cell often there are only a handful of molecules of the same species present. Although particles, in the biological sense, are usually supramolecular assemblies and therefore are composed of a number of molecules, similar methodological principles can be applied to them as to single molecules.

There are at least four areas in which single-molecule or single-particle techniques provide unique gain over ensemble methods. First, individuals can be identified in a crowd and followed in space and time. Considering the unusually dense and crowded environment of the intracellular space, single-molecule and single-particle visualization methods must be applied to uncover the behavior of individual molecular species. Second, the temporal distribution of molecular states may be described; hence stochastic processes, such as the blinking of fluorescent proteins (Dickson et al. 1997), may be identified. Third, the spatial distribution of molecular states may be identified; hence, processes that proceed via parallel pathways, such as protein folding (Dobson and Karplus 1999; Zhuang and Rief 2003), may be explored in detail. Finally, the mechanical properties and functions of biomolecular systems can be unveiled only by single-molecule and single-particle methods, because mechanical force needs to be measured which is a vectorial quantity with a distinct point of action. Biomolecular mechanics entail the investigation of the elastic and viscoelastic properties of biomolecules and the force-generation by mechanoenzymes.

The field of single-molecule and single-particle science evolved in the past 30 years through landmark experiments. Individual actin filaments could be visualized as they glide over a lawn of myosin molecules in what has since become known as the in vitro motility assay (Harada et al. 1987; Kron and Spudich 1986). Myosin (Finer et al. 1994) and kinesin (Svoboda et al. 1993) were the first motor proteins, the mechanical work (force and displacement) of which were measured, by using optical tweezers. Single-molecule mechanical experiments have shown that dsDNA may be overstretched into an S-form, the exact nature of which is still to be uncovered (Smith et al. 1996; Strick et al. 1996). The first protein molecule to be mechanically manipulated, with optical tweezers (Kellermayer et al. 1997; Tskhovrebova et al. 1997) and AFM (Rief et al. 1997a), was the giant muscle protein titin. This mechanical fingerprinting assay has since contributed to the development of a separate methodological field, single-molecule force spectroscopy (Anderson et al. 2013; Dowhan et al. 2015; Fisher et al. 2000; Greene et al. 2008; Lanzicher et al. 2020; Lv et al. 2014; Rief and Grubmuller 2002; Rief et al. 1997b; Ros et al. 2004; Thoma et al. 2018; Zhu et al. 2009). Stretching single RNA hairpins with optical tweezers has shown that RNA can fold against force (Liphardt et al. 2001). It has also been shown that ribosomes are mechanoenzymes that work against applied force via phases of discrete steps and pauses (Wen et al. 2008).

Single-particle approaches have been applied to viruses soon after AFM imaging began making its way into biomolecular sciences. The topography of individual T4 bacteriophage particles was obtained by scanning them, in air, with the AFM (Ikai et al. 1993). Single-molecule mechanical experiments revealed that the portal motor of the φ29 bacteriophage is the strongest mechanoenzyme known to date (Smith et al. 2001) (see also below). AFM-based nanoindentation experiments revealed that viral capsids are resilient nanocontainers (Ivanovska et al. 2007; Ivanovska et al. 2004; Klug et al. 2006; Michel et al. 2006; Roos et al. 2007). The single-particle approach to understanding viral structure, function, and mechanics paved the way towards the emergence of physical virology (Baclayon et al. 2010; Marchetti et al. 2016; Roos et al. 2010).

In the following we address some of the pivotal aspects of the single-particle applications in virus analysis, which may be appropriately called *single-particle virology*. It is important to note that in spite of the amazing progress in the cryo-electron microscopic investigation of viruses, cryo-EM is not discussed here as it relies on class averaging of particle images obtained on frozen virions (Kaelber et al. 2017). By contrast, in single-particle virology, individual virions are studied in their quasi-native, functional environment.

## General structure and life cycle of viruses

The history of virology dates back to the end of the eighteenth century to Edward Jenner’s valiant experiment, which is the first vaccination effort, against smallpox, a highly contagious viral infection (Riedel 2005). The remarkable experiment notwithstanding, the existence of viruses remained unknown for several decades. Although the discovery of the Tobacco mosaic virus by D. Ivanovski (1892) and M. Beijerinck (1898) and bacteriophages by F. W. Twort (1915) and F. d’Herelle (1917) had given experimental evidence for the existence of infectious agents smaller than bacteria (Duckworth 1976), viruses could be visualized only much later, with the introduction of the electron microscope (EM) in 1939 (Goldsmith and Miller 2009). At present, about 80 years after the first visible record of Tobbaco mosaic viruses by EM (Kausche et al. 1939), single-particle methods enable us not only to visualize individual virions but also to study environmental effects such as changes in pH (Wilts et al. 2015), temperature (Vörös et al. 2018), or osmotic pressure (Cordova et al. 2003; Evilevitch et al. 2005; Evilevitch et al. 2003; Jeembaeva et al. 2008) on capsids in real time in quasi physiological aqueous conditions.

Viruses are small obligate intracellular parasites (Gelderblom 1996). They are classified on the basis of morphology, chemical composition, and mode of replication. The complete virus particle is composed of either RNA or DNA genome—single stranded or double stranded, linear or circular—packaged inside a symmetric protein capsid. The entire genome may be formed by a single nucleic acid molecule (monopartite genome) or segments of it (multipartite genome). The different types of genomes lead to different replication strategies. Capsids are formed as single- or double-layered protein shells and consist of only one or a few structural protein species (Lucas 2010). Self-assembly of virus capsids follows two basic patterns: helical symmetry, in which nucleocapsids consist of a helical array of proteins wrapped around a helical filament of nucleic acid, or icosahedral symmetry, in which the protein subunits assemble into a symmetric shell that covers the nucleic acid–containing core. Icosahedral viral capsids need to withstand the high pressure from the tightly packaged DNA. Larger viruses often have a complex architecture consisting of both helical and isometric symmetries confined to different structural components. In enveloped viruses, the nucleocapsid is surrounded by a lipid bilayer derived from the modified host cell membrane decorated with virus envelope glycoproteins. Virus envelopes can be considered additional protective shells. A head-tail morphology is unique to viruses that infect bacteria, which are known as bacteriophages. The head of the virus has an icosahedral shape connected to a helical tail (Hrebík et al. 2019). The phage tail is attached to one of the fivefold vertices of the head in which a pentamer of capsid proteins is replaced by a dodecahedral portal complex. The tails of podoviruses are variable in size and protein composition; however, they share common organizational motifs. The capsids of some phages contain inner core proteins that are associated with the portal complex and play a role during infection. The capsid and envelope protect the viral genome from digestion by nucleases, maintain virion integrity, and play a role in viral infection: they facilitate virus attachment to target cells, the entry into the host, the release of its contents into the cells, and the enclosure of the newly formed viral genome (Roos et al. 2007). Capsid and envelope structure determine the method of viral binding, entry, and exit through the host cell membrane. The tail of bacteriophages contains specialized protein subunits for receptor binding, cell wall degradation, and cell membrane penetration (Lander et al. 2009). The inner core proteins are released together with the phage genome and are speculated to play a role in the delivery of DNA into the cell cytoplasm. Packaging of viral genomes of tailed bacteriophages inside procapsids is powered by an ATP-dependent virus-encoded genome-packaging motor that assembles at the portal vertex (Cardone et al. 2012; Lokareddy et al. 2017; Rao and Feiss 2008; Suhanovsky and Teschke 2015).

## Packaging of the viral genome

**Fig. 1:**
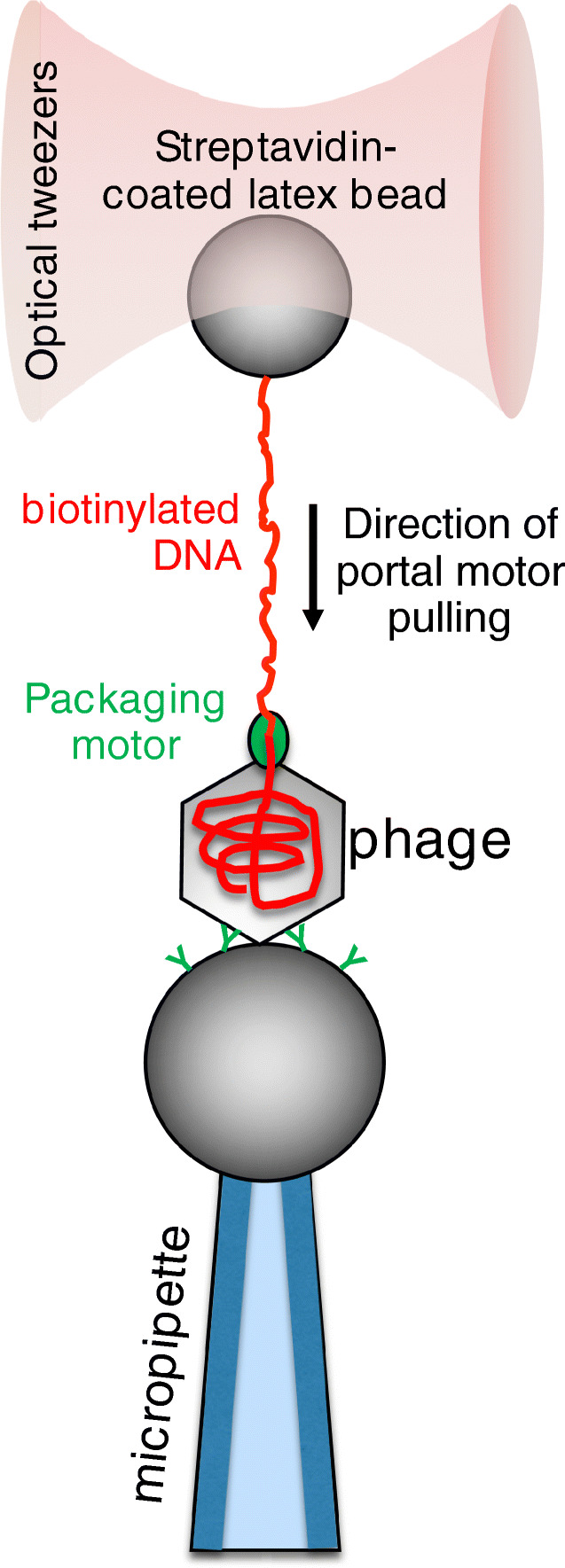
Schematics of measuring the mechanical function of the φ29 bacteriophage portal motor (Smith et al. 2001). The force generated by the DNA-packaging portal motor is transmitted, via the dsDNA molecule, to an optically trapped latex bead attached to the free DNA end, allowing for the measurement of the force. This particular packaging motor stalls at a force of about 60 pN

An essential, first step of the viral life cycle is the tight packaging of newly replicated viral genome into a protein shell, which leads to the emergence of new, infectious virions. Several double-stranded (ds) DNA viruses, including herpes, adenoviruses, and tailed bacteriophages, package their genome into preformed protein procapsids by a nanomotor that is located at its portal complex (Casjens 2011; Rao and Feiss 2008). This molecular motor is an ATP-hydrolyzing DNA translocase which requires chemical energy to condense the typically several-micrometer-long double-stranded DNA into the capsid-confined volume that is approximately 10^−4^ μm^3^ (Sun et al. 2010). In the case of tailed bacteriophages, the diameter of the capsid (30–100 nm) is typically five to six orders of magnitude smaller than the contour length of the viral DNA (3–10 μm), which explains why packaging results in a tightly wound arrangement and near-crystalline DNA density (Tang et al. 2008). To package the highly charged polymer chain into a small confinement, the motor protein has to deliver a significant amount of mechanical work to overcome the increase in entropic, electrostatic, and bending energies of the condensed DNA (Jeembaeva et al. 2010; Tzlil et al. 2003). Single-molecule experiments revealed the nanomechanics of the viral packaging machine, in which the terminus of a partially packaged dsDNA molecule was pulled against the working packaging motor using optical tweezers. In case of the most excessively studied ϕ29 bacteriophage, these measurements revealed that the DNA-packaging motor is processive, can insert DNA into the procapsid at rates of ∼ 100 bp per second, and generates forces up to ∼ 60 piconewtons (Fig. 1) (Smith et al. 2001). The unprecedented high value of forces that single viral packaging motors were able to exert in these experiments makes them the strongest molecular motors reported to date. The speed of packaging decreased with the procapsid filling, possibly because of the increasing internal pressure. This suggests that the high forces generated by the motor are required to package DNA against the increasing internal capsid pressure that can reach up to ∼ 6 MPa by the end of the process (Evilevitch et al. 2003). The ϕ29 motor translocates the DNA in steps of 10 bps that in turn consists of four rapid, non-integer 2.5-bp sub-steps per ATP hydrolysis (Moffitt et al. 2009). The four non-integer steps together with the presence of five motor subunits reveals an asymmetry that demands new models for motor-DNA interactions (Chistol et al. 2012). It has been shown that the ϕ29 motor also rotates the DNA during packaging, and the rotation per base pair increases with filling and leads to a reduction in the motor’s step size as the level of packaging increases (Liu et al. 2014). The packaging of T4 and λ phages measured with optical tweezers was also found to be driven by a very strong (stall force > 60 pN) and processive motor, suggesting that these are universal properties of all dsDNA viral motors that need to package DNA to high density. The only difference that was found in case of T4 and λ motors is that the packaging rates were significantly faster (1–2 kbp per second). Given that the genome of T4 and λ phages is several-fold longer than that of ϕ29, these findings suggest that viral motors scale their packaging speeds according to the genome size (Fuller et al. 2007a; Fuller et al. 2007b).

## Topographical structure of individual virus particles

Viruses assemble spontaneously from their proteinaceous building blocks into a few main structural classes with a variety of sizes and molecular detail. In recent years cryo-electron microscopy has become a key method in revealing viral structure, down to the atomic resolution (Kaelber et al. 2017). However, beyond doubt, the most essential method in investigating the structure of individual viral particles under ambient conditions is the atomic force microscope (AFM) (Allison et al. 2010; Baclayon et al. 2010; de Pablo 2018; de Pablo and Schaap 2019; Kuznetsov and McPherson 2011; Santos and Castanho 2004). Shortly after its invention (Binnig et al. 1986), AFM was applied to imaging viruses, initially in air (Ikai et al. 1993; Valle et al. 1996), then under aqueous buffer conditions (Müller et al. 1997; Ohnesorge et al. 1997). In the decade that followed, AFM has been applied to describing the surface structure of a vast array of different viruses (Chen 2007a, b; Drygin et al. 1998; Dubrovin et al. 2007; Ferreira et al. 2008; Hards et al. 2005; Huff et al. 2004; Kiselyova et al. 2003; Klem et al. 2003; Kuznetsov et al. 2008; Kuznetsov et al. 2005a; Kuznetsov et al. 2005b; Kuznetsov et al. 2004; Kuznetsov et al. 2000; Kuznetsov et al. 2007; Malkin et al. 1999; Malkin et al. 2003; Mat-Arip et al. 2001; Matsko et al. 2001; Moloney et al. 2002; Negishi et al. 2004; Nettikadan et al. 2003; Schmatulla et al. 2006; Trindade et al. 2007). Although in its standard operation the AFM provides a high-resolution topographical image of the sample, by now its applications have expanded to nanomechanics, nanomanipulation, measurement of interactions, and recording of time-dependent processes.

**Fig. 2:**
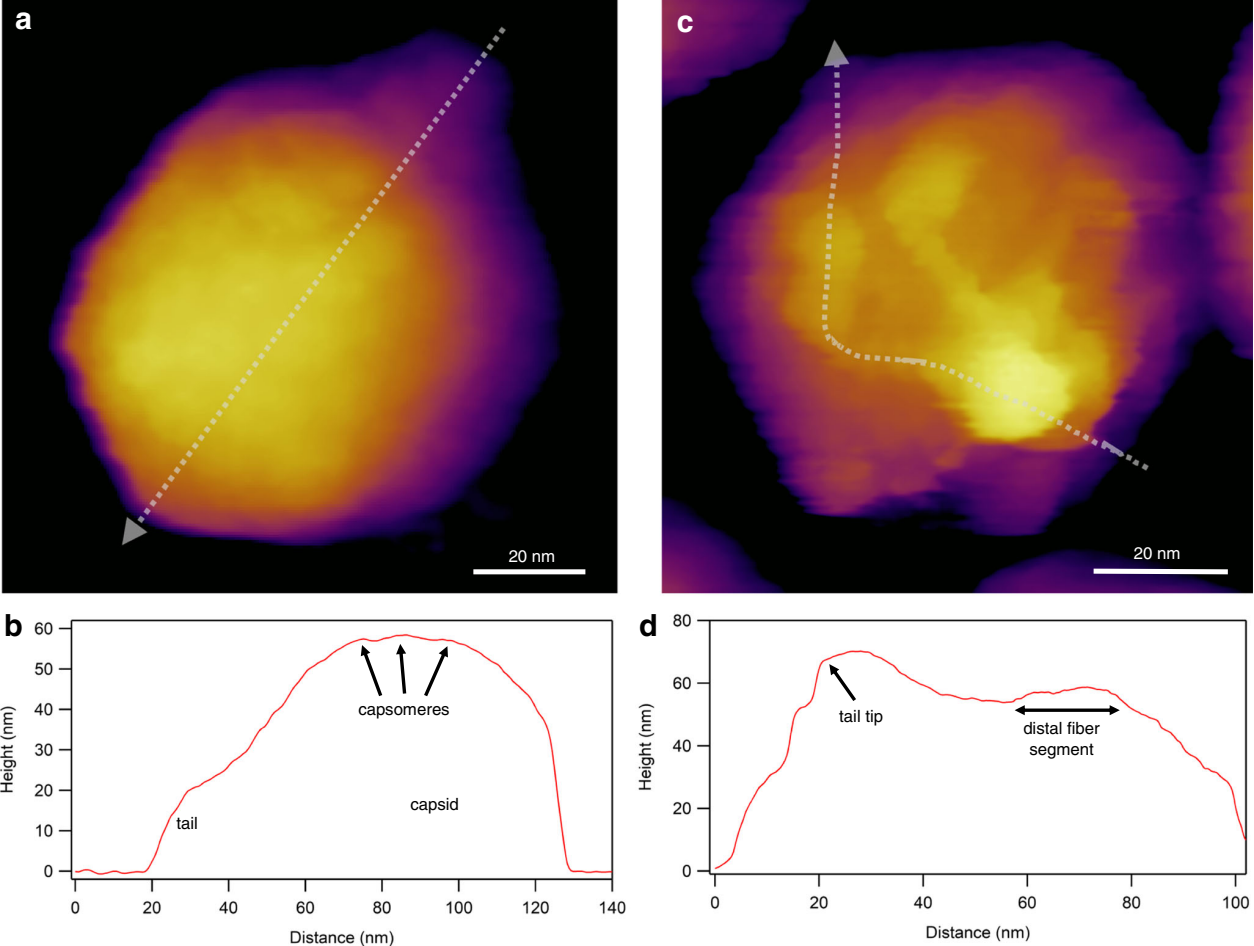
Imaging virions with atomic force microscopy (AFM) in non-contact mode, by using photothermal excitation to resonate the cantilever. **a** Height-contrast AFM image of a T7 bacteriophage particle attached to poly-l-lysine-coated mica. Based on the surface topography, this icosahedral virion is facing towards the buffer solution (phosphate-buffered saline, PBS) with its 3-fold symmetry axis. Individual capsomeres can be discerned in the image. **b** Topographical height profile plot along the axis of the particle image (indicated by the dotted straight line). **c** Height-contrast AFM image of a T7 bacteriophage particle pointing towards the buffer solution with its tail. To immobilize the tail fibers, the sample was chemically fixed with 2.5% glutaraldehyde and imaged in PBS. **d** Topographical height profile plot along the axis of a tail fiber (indicated by the dotted freehand line)

**Fig. 3:**
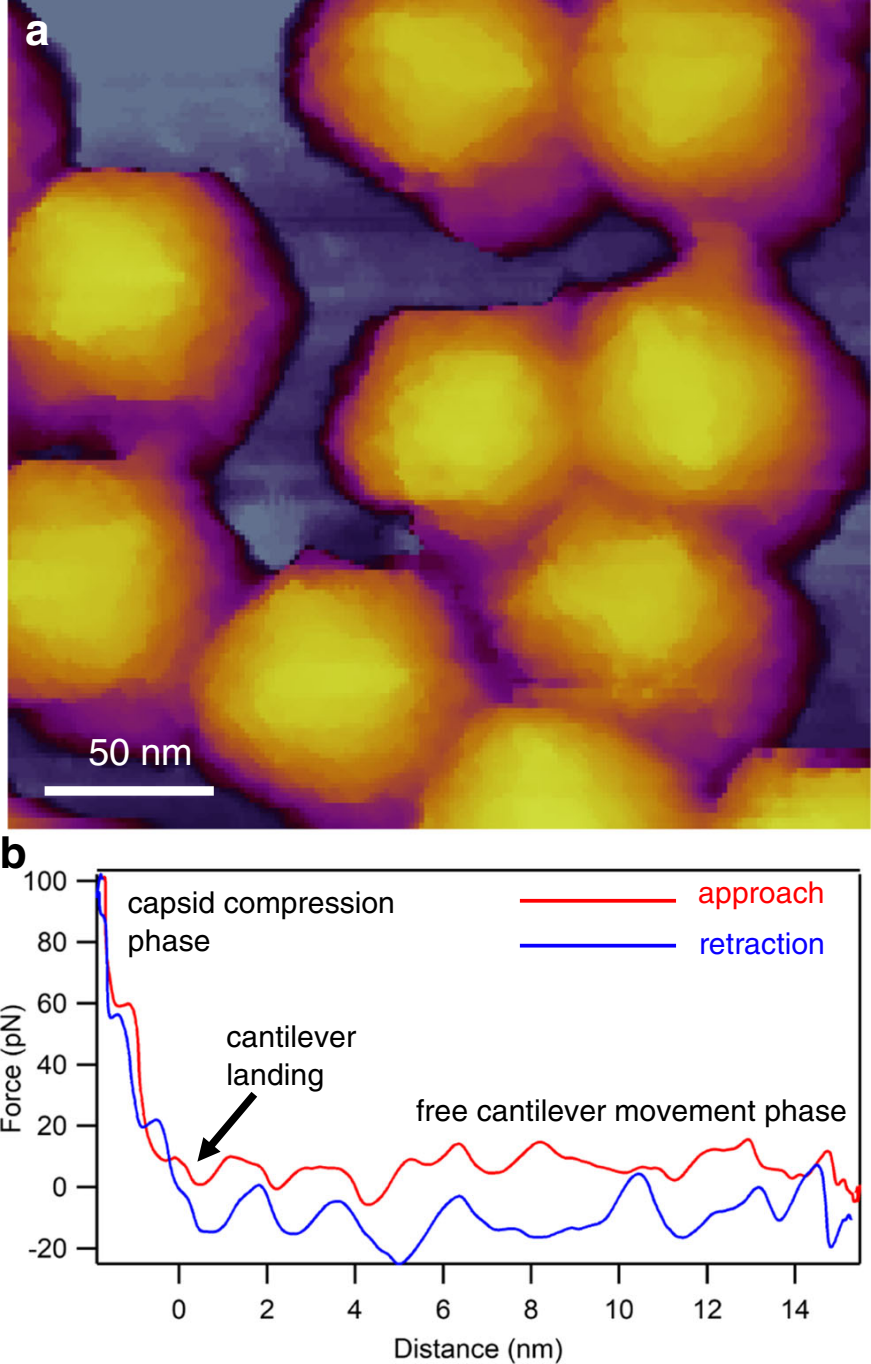
Imaging virions with AFM in jumping mode (fast force mapping). **a** Height-contrast AFM image of a T7 bacteriophage particle attached to poly-l-lysine-coated mica. **b** Example of a force versus distance curve that lies behind every pixel of the FFM image

In an AFM the sample is scanned with a sharp tip at the end of a flexible cantilever. During scanning the tip is brought in close proximity with the surface that causes the cantilever to bend (de Pablo 2018). The bending of the cantilever, exerted by the forces acting between the tip and the surface, is detected through the deflection of a laser beam reflected from the back of the cantilever (de Pablo and Schaap 2019). Usually deflection is kept stable by a feedback loop, meaning that it is held at constant distance from the sample (de Pablo and Schaap 2019). A commonly used gentle imaging mode of soft biological samples is “tapping” or “amplitude-modulation” mode, in which the direct contact between the tip and the sample is minimized (Kuznetsov and McPherson 2011) (Fig. 2). Resonating the cantilever tip without shaking the entire cantilever base, such as in photothermal excitation, for example, provides a particularly stable imaging technique. Besides height contrast (Fig. 2a, c), additional contrast mechanisms (e.g., amplitude, phase) provide further insight into local structural and mechanical (elasticity, viscosity) properties of the virion. Considering that each pixel of a height-contrast image contains topographical height information, height profile plots may be obtained along axial (Fig. 2b) or arbitrary (Fig. 2d) directions over the capsid surface. It is important to emphasize that the surface topographical image obtained after scanning the sample is not an ensemble average but is characteristic of the individual virion. In spite of collecting an image based on a single nanoscale object, amazing structural detail can be revealed (*c.f.* the tail fiber domain structure in Fig. 2c). An AFM imaging mode that has become popular in viral analysis is “jumping mode” or fast force mapping (FFM) (Fig. 3). In FFM imaging the cantilever is moved up and down (sinusoidally) with a frequency (~ 50–300 Hz) much below the cantilever’s resonance frequency. There is one oscillation cycle for every pixel of the image. In each oscillation cycle a force versus distance plot is obtained which corresponds to a nanoindentation-retraction trial (Fig. 3b). In FFM mode the forces which the virion is exposed to are better controlled than in tapping mode. Furthermore, topography, adhesion, and elasticity maps may be calculated from the force traces. Finally, resonant modes which a functional virus might respond to (Kellermayer et al. 2018) are avoided. Altogether, the functions of AFM provide much more than surface topography. High-resolution force versus distance curves derived from indentation and pulling experiments give information about the detailed nanomechanical properties of capsids (Marchetti et al. 2016) (see below). The Young’s modulus, stiffness, and rupture forces are directly associated with capsid stability (de Pablo 2018). Combining AFM imaging with nanoindentation allows to map the structural consequences of the mechanical perturbations. By utilizing this approach, the mechanical fatigue, the self-healing capacity, and the mechanically induced partial disassembly of capsids could be uncovered (Ortega-Esteban et al. 2013; Valbuena and Mateu 2015). Enveloped viruses such as HIV (Rankovic et al. 2017) and SARS-CoV (Lin et al. 2005) may be more challenging to investigate with AFM due to the dynamics of the surface coating. In the case of isolated HIV-1, it was found that the viral uncoating process depends on the stage of reverse transcription (Rankovic et al. 2017).

A unique feature of AFM lies in its function to follow dynamic processes even in more complex biological systems, such as on the surface of the living cell. Hence, virus-cell interactions, viral budding or disassembly can be followed in an unlabeled environment (Baclayon et al. 2010; Kiselyova et al. 2003). SARS-CoV virions were shown to bud and rupture the plasma membrane, assisted by the underlying actin cytoskeleton in their transport. Individual retrovirus budding through the plasma membrane of living cells infected with Moloney murine leukemia virus was monitored in real time with AFM, wherein two kinetically distinct pathways were observed (Gladnikoff and Rousso 2008). HIV budding was registered over time by measuring the viral protrusion height on the surface of infected cell membranes (Gladnikoff et al. 2009). Finally, thanks to the rapid development of image processing, automatic classification methods for single virus discrimination based on AFM imaging can be constructed, which might potentially be important in diagnostic applications. Blocklitz el al. investigated the maximal height, volume, and occupied area of five different virus species (Varicella-zoster virus, Porcine teschovirus, Tobacco mosaic virus, Coliphage M13, and Enterobacteria phage PsP3) on AFM images, and designed an automatic image classification method with an identification accuracy over 95% (Bocklitz et al. 2014).

## Viral capsid nanomechanics

**Fig. 4:**
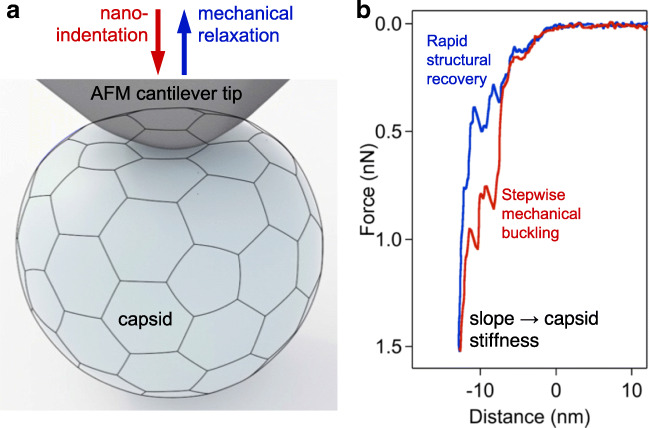
Nanoindentation of a viral capsid. a Schematics of the experiment. The capsid and AFM cantilever tip are indicated with a realistic relative scale. Mechanical information can be collected, in the form of force versus distance data, during both the indentation and retraction phases of the experiment. **b** Force versus distance plot obtained on a T7 phage particle (Vörös et al. 2017). Force sawteeth during indentation and retraction point at buckling and unbuckling events, respectively, which result in stepwise, 6-nm reversible structural changes in the capsid. The slope of the indentation trace may be used to calculate the stiffness of the capsid

Besides being a powerful imaging technique, AFM also provides a possibility for exploring the mechanical properties of viral capsids. In the recent past, single-particle nanoindentation experiments enabled the characterization of the physical properties of viral capsids with unprecedented detail and insight (Carrasco et al. 2011; Castellanos et al. 2012; Hernando-Pérez et al. 2014b; Ivanovska et al. 2011; Roos et al. 2012; Snijder et al. 2012). In a nanoindentation experiment, the tip of the AFM cantilever is lowered on the surface of a substrate-bound capsid until a pre-adjusted force is reached by the bending of the cantilever (Fig. 4a). Then, the cantilever is retracted. Force, obtained from the bending of the calibrated cantilever, is recorded as a function of cantilever displacement (Fig. 4b). Nanoindentation results showed that many virus protein capsids behave like elastic and robust nanocontainers (Snijder et al. 2013). Furthermore, below a certain force threshold, the capsids behave as elastic nanoshells, whereas at higher forces material failure occurs and the capsid collapses. The AFM has also been used to apply well-controlled forces to single capsids to trigger disintegration. For adenovirus, tip penetration causes the same sequence of events as the uncoating in vivo, starting with the release of pentons, followed by capsid disruption (Ortega-Esteban et al. 2015; Ortega-Esteban et al. 2013). Based on AFM imaging subsequent to the nanoindentation experiment, it was found that after the mechanical rupture of the capsid the viral core of a mutant adenovirus (TS1) remained visible as a condensed blob, whereas the core of the wild-type virus could not be resolved. AFM and single-molecule fluorescence microscopy were combined to specifically observe genome uncoating from wild-type and TS1 adenovirus (Cordova et al. 2014). Viruses that infect eukaryotic cells usually undergo structural changes leading to complete capsid disassembly and release of the viral genome (Mateu 2011; Wilts et al. 2015). Conformational transitions in the capsid can be triggered by mechanical cues. HIV-1 undergoes a protease-mediated maturation process, which is necessary for successful infection. By using AFM, it was discovered that HIV undergoes a “stiffness switch,” which is a dramatic reduction in particle stability during maturation mediated by the viral envelope protein (Pang et al. 2013). High-resolution AFM nanoindentation experiments on DNA-filled T7 bacteriophages revealed that the elastic region of the force curves contained discrete, stepwise transitions (Fig. 4b). These transitions lead to capsid buckling in steps, the size of which is integer multiples of about half a nanometer. The transitions are reversible, as similar steps were observed during cantilever retraction. The reverse steps contribute to the structural recovery of the capsid following mechanical perturbation. The steps were present even after DNA removal, indicating that they reflect the structure and dynamics of the capsid proteins (May and Brooks Iii 2012; Vörös et al. 2017). Upon gently tapping the capsid wall of the T7 bacteriophage with the tip of an AFM cantilever, the virus rapidly ejected its DNA. At increasing mechanical loads, the rate of triggering DNA ejection increased exponentially. The low forces employed caused very small changes in the internal pressure of the capsid, yet they were sufficient to trigger DNA ejection. Thus, a DNA-filled capsid is in a state poised for expelling its genomic material and the proteins required for the faithful execution of the initial steps of phage infection (Kellermayer et al. 2018). Nanomechanical measurements may reveal the response of the virion to environmental factors. Exposing T7 bacteriophage to a thermal treatment at 65 °C caused DNA release due to the tail complex breaking off from the capsid. The loss of DNA and/or thermally driven changes in capsid protein structure results in reduced capsid stiffness and breaking force. Further heating to 80 °C leads to the appearance of large globular particles that likely correspond to disassembled capsids. It also results in partial structural stabilization of the remaining capsids, most likely caused by rearrangements within the capsid wall (partial denaturation of the component gp10A proteins). Even though the capsids are destabilized, they are still able to withstand high temperatures with a more or less intact global topographical structure (Vörös et al. 2018). Altogether, AFM-based nanomechanical experiments provide a sensitive tool to explore the properties of viruses (Cieplak and Robbins 2013; Hernando-Pérez et al. 2014a; Kurland et al. 2012; Mateu 2012). Nanomechanical parameters, such as stiffness and capsid breaking force, may reveal molecular mechanisms underlying capsid maturation and the packaging, storage, and release of genetic material. Combining AFM with other methods, such as total internal reflection fluorescence (TIRF), provides further possibilities for the complex analysis of viral biological processes.

## Viral infection tracking by super-resolution single-molecule fluorescence imaging

**Fig. 5:**
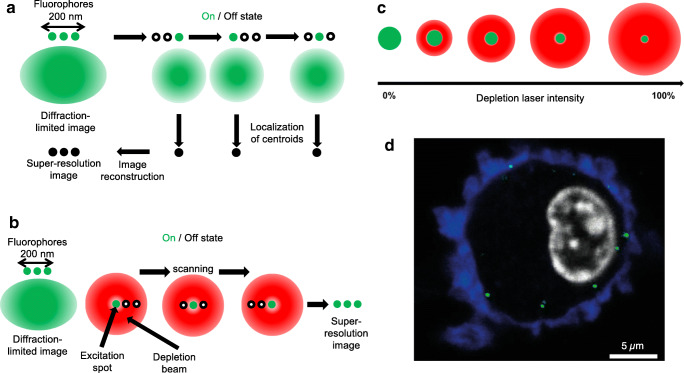
Super-resolution microscopy techniques employed in single-particle virus research. **a** Principles of stochastic methods (STORM and PALM). **b** Principles of the deterministic approach in STED. **c** Principle of the shaping of the point spread function (PSF). **d** Example of influenzavirus A (IAV) infection in a human dendritic cell. Blue color corresponds to Alcian blue in the cellular environment, gray to the DAPI-stained nucleus, and green to IAV nucleoprotein labeled with FITC-conjugated antibody. Adapted from (Baharom et al. 2017)

Fluorescence microscopy techniques have long been widely used for studying various biological processes due to their versatility. Although wide-field fluorescence microscopy is most common due to ease of use and relatively low cost, its significant drawback is that photons from out-of-focus regions contribute to the observed signal; therefore, the imaging of small particles such as viruses or proteins is challenging due to the high background signal. Special forms of fluorescence microscopy have been developed to overcome this problem. In total internal reflection fluorescence (TIRF) and confocal laser scanning microscopy (CLSM), light is detected only from the focal plane; thus, the low background allows the studying of individual molecules in small (TIRF) or large (CLSM) volumes even with three-dimensional reconstruction. These techniques are, however, limited by the diffraction of light, and the best resolution achieved is ~ 200 nm according to Abbe’s law. Biomolecular processes occurring in this length scale, e.g., virus-host cell binding or enzyme-substrate interaction, thus have not been possible to explore with optical microscopy. One solution to this problem was Förster resonance energy transfer (FRET) which can be used as a molecular ruler across small distances (1–10 nm). Despite its technical challenges, FRET has been applied to investigate virus-host interactions (Emmott et al. 2015; Koh et al. 2011; Takagi et al. 2017). A fundamental solution that overcomes the limitations of Abbe’s law is super-resolution (SR) microscopy, which has been revolutionizing life sciences and is paving its way into single-particle virology. SR microscopies can be divided into two broad groups based on their approach to bypass the diffraction limit: stochastic (Fig. 5a) and deterministic (Fig. 5b).

Stochastic SR microscopies are based on either photoactivatable (photoactivated localization microscopy, PALM) or photoswitchable (stochastic optical reconstruction microscopy, STORM) dyes (Betzig et al. 2006; Rust et al. 2006). During image acquisition, only a small subset of the labeled particles is activated simultaneously by the excitation laser; then, the dye molecules are bleached. The laser activates the fluorophores with a spatial stochasticity until all of them have emitted photons and become bleached (Fig. 5a). Emission is detected by sensitive sCMOS detectors, and the centroids of the fluorescence spots are calculated based on the point spread function (PSF). Using these coordinates, super-resolution images are reconstructed, the typical spatial resolution of which may be as good as 20 nm. By contrast, the temporal resolution is low due to the numerous activation/photobleaching cycles necessary for image formation. PALM microscopy was used in a proof-of-concept effort to follow single virus particles in transfected cells. It was clearly demonstrated that the imaging speed can be sufficient to reconstitute trajectories of single virus particles in live cells (Manley et al. 2008). Several studies on influenza hemagglutinin (HA) protein were carried out by PALM microscopy variations revealing HA’s relationship with host-cell actin meshwork. These results may help to find new targets to develop antiviral treatments (Gudheti et al. 2013; Hess et al. 2007; Nelson et al. 2014). STORM microscopy was used in several studies related to viral infection. Pereira et al. proved that the HIV-1 matrix shell and capsid core can be quantified by STORM. It was also demonstrated that HIV particles undergo dramatic rearrangement immediately after entry into the target cells (Pereira et al. 2012). A further study combining STORM and cryo-EM revealed that this increase in size is solely triggered by the CD4-Env binding and is independent of virus fusion (Pham et al. 2015).

Deterministic SR microscopies (Fig. 5b) rely on the controlled excitation of fluorophores in the focal volume and the confinement of the excitation volume by point-spread-function engineering (Fig. 5c). The most representative member of this category is stimulated emission depletion (STED) microscopy (Hell and Wichmann 1994). STED uses selective deactivation of fluorophores with a doughnut-shaped depletion laser beam, which creates a minimized excitation area at the focal point. The size of this area can be reduced by increasing the intensity of the depletion laser (Fig. 5c), yielding a much smaller focal point than would be allowed by the diffraction limit. STED is combined with point-scanning devices; thus, photon collection is deterministic and SR image formation is immediate. As a result, temporal resolution exceeds that of stochastic approaches and there is no need for image post-processing. Due to the fast imaging rate, STED microscopy is suitable for live-cell imaging and hence the investigation of viral entry into the cell (Fig. 5d). The resolution of STED is theoretically infinite; however, it depends strongly on the fluorophores and the hardware used. The typical resolution that can be achieved even in live-cell imaging is 30–40 nm. STED microscopy related to viruses was first demonstrated with GFP-labeled rotaviruses (Willig et al. 2006). STED microscopy was used to explore how HIV-1 enters CD4+ cells. It was clearly demonstrated, by using dual-color STED microscopy, that cell contact can induce the clustering of mobile Env molecules promoting the maturation of the virion (Chojnacki et al. 2012). STED-FCS measurements have confirmed that Env mobility is dependent on the virus maturation status (Chojnacki et al. 2017). Recently, a novel super-resolution approach called MINFLUX was developed with a capability of resolving luminous points within a 1–3-nm range even in live cells in three dimensions (Balzarotti et al. 2017; Gwosch et al. 2020), raising the possibility of following viral assembly in situ. Since subviral details may be resolved, yet the speed of image formation is sufficient for tracking single particles in live cells, SR microscopies will likely play an important role in unraveling the molecular details of the viral life cycle. Although key parameters such as temporal and spatial resolution need to be further improved, super-resolution microscopies are expected to expand and contribute significantly to understanding the viral infectious cycle at the level of the single virion.

## Mechanisms and mechanics of viral infection

**Fig. 6:**
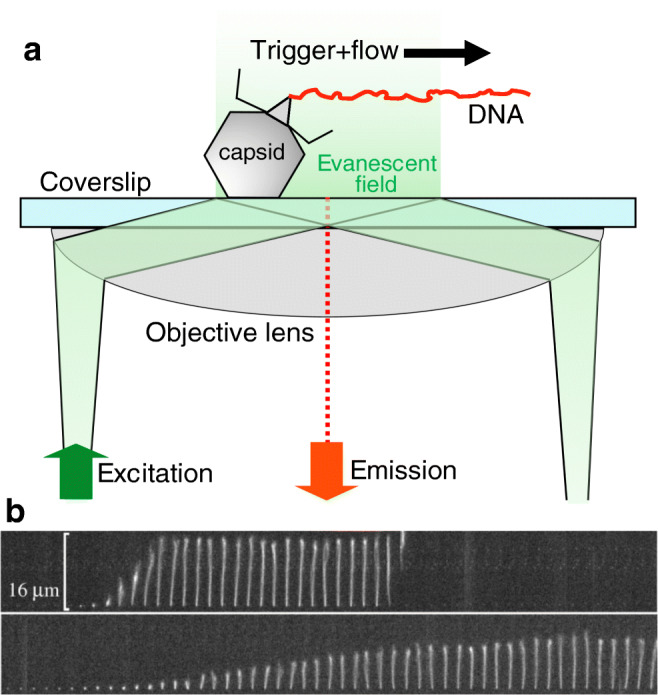
**a** Schematics of investigating the genomic DNA release from individual phage particles by using a microfluidic device, total internal reflection fluorescence (TIRF) microscopy, and DNA-intercalating fluorophores. **b** Time-resolved images of the release of dsDNA from a single λ-phage particle. DNA ejection was triggered by adding LamB (maltoporin), an *E. coli* outer-membrane protein, and the DNA molecule was visualized by rapid staining with the fluorescent dye SUBR Gold present in the buffer solution of the microfluidic chamber. Time delay between consecutive DNA images is 0.25 s. Upper and lower image series were recorded in the presence of 10 mM NaCl and 10 MgSO_4_, respectively. Adapted from (Grayson et al. 2007)

Ever since the discovery of viruses, the mechanisms of the viral infection process have been in the center of scientific, medical, and even economic interest. Most of our knowledge has come from the high-resolution structures provided by electron microscopy studies of the past decades, which enabled us to predict the functions of different parts of viral nanomachines. In the recent past, cryo-electron microscopy has been providing an ever-increasing detail about the structure of viruses, lending clues to the infection mechanisms (Guo et al. 2014; Kaelber et al. 2017; Pham et al. 2015; Serwer et al. 2018; Shingler et al. 2013; Wrapp et al. 2020). Besides imaging methods, bulk assays relying on molecular biological techniques have provided key elements to understanding the steps of viral infection. EM and molecular biology techniques provide ensemble snapshots of the viral infection process, but the continuous timeline of events related to a single virion largely remains hidden. Different types of viruses (e.g., DNA and RNA viruses) use vastly different tactics to invade the host. In the recent past, remarkable experimental observations were made on the dynamics of the first steps of infection by individual DNA-virus particles. The ejection of genomic dsDNA was followed in real time by using a combination of microfluidics and total internal reflection fluorescence (TIRF) video microscopy (Grayson et al. 2007; Mangenot et al. 2005) (Fig. 6). In the experimental layout, virus particles are attached to a coverslip surface which serves as the bottom of a microfluidic device (Fig. 6a). The viruses are activated by injecting the relevant activator protein along with DNA-intercalating dyes. The interaction of phages and their protein receptors resulted in the sudden ejection of DNA molecules which were immediately stretched out by the flow, allowing their length to be measured as a function of time. In the case of T5, rapid DNA ejection steps were observed with intermittent pauses at distinct locations along the genome, which correlated with the positions of genetically engineered DNA nicks (Mangenot et al. 2005). This mechanism appears to be unique compared with other viruses. In the case of λ phage, for example, DNA release was continuous with no apparent pauses (Grayson et al. 2007). λ-DNA ejection kinetics is strongly influenced by cations in the ejection medium (Fig. 6b), which is similar to the effect of environmental osmotic pressure changes (Casjens and Hendrix 2015; Marion and Šiber 2014; São-José et al. 2007; Wu et al. 2010). Similar experimental approach was used to monitor the ejection of DNA from the Archaeal virus His1. The rapid DNA ejection process of His1 was dependent on cation concentrations and osmotic pressure changes, similarly to bacteriophages (Hanhijärvi et al. 2013). By contrast, His1 DNA ejection was insensitive to changes in pH and temperature, explaining why this virus can withstand harsh environmental conditions (Hanhijärvi et al. 2016). The major, yet-to-be answered question concerns the energetics of DNA ejection. It is thought that the initial forces driving the ejection are stored in the self-repulsion between tightly packaged DNA segments. However, this energy cannot be the sole driving force for complete ejection. There needs to be an additional force that finalizes DNA translocation across the target membrane, which might be contributed by internal host enzymes (Molineux and Panja 2013).

Intact phages may be labeled via their DNA with dye amounts so low that their presence does not disrupt their functions (Eriksson et al. 2007). By making use of this method, λ phages were labeled and individual DNA injections into *E. coli* were successfully observed (Van Valen et al. 2012). DNA translocations, intermittent with pauses, were complete only by 5 min. By contrast, in vitro DNA ejection proceeds continuously and becomes finished within 10 s (Grayson et al. 2007) (Fig. 6b). In vitro studies showed that ejection velocity is controlled by the amount of DNA left inside the capsid, whereas in vivo translocation is additionally governed by forces acting on the DNA that is already inside the target cell. Further techniques for tracking and visualizing viruses during their infectious steps include patch-clamp methods which have been employed to track the docking of λ phages to their target receptors incorporated in a supported lipid bilayer (Gurnev et al. 2006); holographic microscopy, which has been used to track the orientation and DNA release of bacteriophage λ (Goldfain et al. 2016). The average DNA release measured by this method was close to that obtained in in vitro experiments using fluorescent labeling. Altogether, to reveal the molecular mechanisms of the numerous different tactical processes that viruses have devised for infecting the host organism, a combination of methods that allow to monitor the spatial and temporal dynamics and mechanics of the different viral components is required.

## Conclusion and perspectives

Viruses are amazing nanoscale machineries which, in spite of their miniscule size and relatively low complexity, are capable of invading the host organism with a puzzling array of tactical mechanisms. Interest in understanding how viruses replicate, assemble, and infect has never vanished ever since their discovery. Single-particle imaging and manipulation methods are of unequalled importance in unveiling the mechanistic detail behind the infectious cycle of viruses. Considering the technological pressures in the recent SARS-CoV-2 pandemia towards understanding, diagnosing, treating, and preventing coronavirus infection (Al-Qahtani 2020; Astuti and Ysrafil 2020; Sheng et al. 2020; Wang et al. 2020; Yan et al. 2020a; Yan et al. 2020b; Zhang et al. 2020) and viral infections in general, single-particle virology may provide a unique edge in combating viral diseases.

## Data Availability

Not applicable.
